# Comparative liver proteome analysis of feedlot steer calves reveals growth trait-specific pathways influenced by calving season

**DOI:** 10.1186/s40104-026-01370-6

**Published:** 2026-04-03

**Authors:** Chris Johnson, Amit Singh, Zahid Hasan, Kamrul Islam, Zhibo Yang, Mindy King, Andrew Foote, David Lalman, Nagib Ahsan, Paul Beck

**Affiliations:** 1 Animal & Food Sciences, Oklahoma State University, Stillwater, OK 74078 USA; 2 Department of Chemistry and Biochemistry, University of Oklahoma, Norman, OK 73019 USA; 3 Department of Biochemistry and Physiology, University of Oklahoma Health Sciences Center, Oklahoma City, OK 73104 USA; 4 Department of Animal Science and Industry, Kansas State University, Manhattan, KS 66506 USA; 5 Mass Spectrometry, Proteomics and Metabolomics Core Facility, Stephenson Life Sciences Research Center, University of Oklahoma, Norman, OK 73019 USA

**Keywords:** Birth season, Bovine liver, Feedlot performance, Growth potential, Proteomics

## Abstract

**Background:**

The bovine liver is a key organ governing nutrient metabolism, immune regulation, and growth. However, the effects of birth season and growth potential on hepatic protein expression remain poorly understood.

**Results:**

This study investigated the liver proteome of steer calves born during the spring and fall 2023 calving seasons (*n* = 5–6 steer/growth trait/season). Using a comparative label-free quantitative proteomics approach, 2,133 proteins were identified and quantified following feedlot entry. Principal component and hierarchical clustering analyses revealed distinct segregation of protein expression profiles according to both birth season (spring vs. fall) and growth trait (high vs. moderate), with calving season exerting the stronger overall influence. Bioinformatic and pathway enrichment analyses identified significant growth- and season-independent proteins. Growth-associated proteins were primarily involved in immune signaling, including antigen processing and presentation, as well as glutathione-CYP detoxification pathways. In contrast, season-independent proteins were enriched in pathways related to circadian rhythm, hormonal regulation, and muscle contraction.

**Conclusion:**

These results demonstrate that both growth trajectory and season of birth independently modulate the bovine liver proteome, with stronger seasonal effects, providing novel insight into the metabolic and immune mechanisms underlying variation in calf growth performance.

**Supplementary Information:**

The online version contains supplementary material available at 10.1186/s40104-026-01370-6.

## Introduction

Proteomics is an emerging discipline within animal science capable of quantifying the proteome in complex tissues; however, its application has been neglected because of cost and lack of awareness of the potential of this technology by animal scientists [1]. Previous studies have emphasized the importance of proteomics in understanding the quality of food products and post-harvest protein alterations [2]. Yet, as global beef consumption continues to rise [3], the industry is operating with historically low cattle inventories due to prolonged drought and declining rangeland resources [4]. Broader implementation of proteomic technologies could enable researchers to identify divergent biological pathways associated with production traits, evaluate the influence of environmental stressors beginning in early life, and ultimately guide more informed genetic selection strategies. Proteomic approaches are uniquely suited to detect persistent molecular signatures of early-life environmental and developmental stress that may not be evident at the phenotypic level.

The limitation of natural forage availability in semiarid environments has the potential to result in unfavorable outcomes of progeny growth and development [5–7]. Maternal nutrient intake during gestation can alter progeny calf health and performance [8–10]. Cattle with low weaning weights grew more rapidly during backgrounding and at a similar rate in the feedlot, resulting in more rapid growth overall from weaning to 30 months of age [11]. Tudor and O’Rourke in 1980 observed that severe nutritional restriction from birth to weaning resulted in limited growth, whereas concentrate-based (high-energy) feeding from weaning to harvest resulted in greater fat thickness at the same live and carcass weights compared with cattle well-nourished prior to weaning [12]. Calves born in Iowa classified as being born following a wet year had greater body weights (BWs) at feedlot entry and reduced number of days on feed (DOF) when compared to calves born following a dry year [13]. Calves born in the fall season often experience late gestational heat stress in utero, which has been reported in dairy calves to compromise fetal growth and alter neonatal metabolism and immune function [14]. Fall-born calves were also observed to have slightly reduced skeletal, muscle, and organ growth that contributed to reduced total birth weights, rather than disproportionate growth [15].

The liver is constantly exposed to a wide variety of bacterial products, environmental toxins, and food antigens. The liver plays a major role in the innate immune response, the induction of immune tolerance, hematopoiesis during fetal development, and it serves as one of the first lines of defense between the host and the external environment [16, 17]. To efficiently and rapidly protect against potentially toxic agents without generating an energetically costly immune response, the liver depends on its strong innate immune response. Hepatocytes, the parenchymal cells of the liver, are responsible for producing 80% to 90% of circulating innate immunity proteins, and the liver contains a large number of resident immune cells [17]. Liver-mediated immune tolerance requires the complex interaction of hepatocytes, liver nonparenchymal cells, and immune cells [17]. Hepatocytes robustly express and release large amounts of proteins in the blood, such as acute phase proteins, and rely on pathogenic and inflammatory signals to release a variety of innate immune proteins into the bloodstream to combat disease [17]. Many of these early-life environmental effects converge on the liver, a central organ integrating immune function, metabolism, and growth regulation.

In addition to the liver’s immunologic roles, the liver serves as a central hub for nutrient and lipid metabolism [18], which indirectly promotes growth by affecting feed conversion efficiency [19–21]. Low feed efficient cattle were reported to have upregulated expression of fatty acid synthase and other genes important for lipid metabolism [18, 21] that were altered from high feed efficient cattle. Maternal nutrient supply to the neonate can create long-term alterations in liver development and functionality [22], and create subsequent limitations on lipid metabolism and glucose metabolism, which causes insulin sensitivity to alter [21].

Therefore, the objective of this study was to characterize growth- and season-associated remodeling of the bovine liver proteome in spring- and fall-born steer calves with divergent growth potential following feedlot entry, using a label-free quantitative proteomics approach. By identifying season- and growth-specific molecular pathways, this work provides novel mechanistic insight into how early-life environmental conditions and genetic growth potential influence metabolic efficiency, immune regulation, and liver-mediated growth performance.

## Materials and methods

All animal procedures used in this experiment were approved by the Institutional Care and Use Committee (IACUC-21-60) of Oklahoma State University.

### Cattle

The Oklahoma State University Range Cow Research Center (OSU RCRC) consists of two closed cow herds that each utilize a spring (South Range Unit) and fall (North Range Unit) calving season. Throughout the year, cows grazed seasonally available native range consisting of primarily tallgrass prairie species and were supplemented to meet their production phase nutrient requirements. These herds have been managed similarly for the last 12-year with shared bulls and similar artificial insemination (AI) management resulting in cowherds with similar genetic backgrounds. The cow herds consisted of commercial beef cows of mixed British-type breeding, which were bred to purebred Angus sires using standardized AI protocols. Only male offspring (steer calves) were enrolled in the experimental design; female calves were excluded prior to weaning and were not included in any performance or proteomic analyses. Synchronization for AI breeding for both Range Units followed the same 14-d controlled internal drug release (CIDR)- prostaglandin and timed AI for primiparous cows and a 7-d Co-Synch with CIDR breeding synchronization protocol as described by Larson et al. [23].

The experimental design evaluated the effects of sire expected progeny difference (EPD) growth trait and season of birth on liver proteomic profiles. Growth traits were determined by sire selection using yearling weight (YW) EPD values. Purebred Angus sires were selected to produce offspring that were classified as either high growth (Hig) or moderate growth (Mod). Sires in the Hig category had a mean YW EPD of 72 ± 5.4 kg, whereas sires in the Mod category had a mean YW EPD of 43 ± 3.2 kg, based on individual sire EPDs reported to the American Angus Association as of December 22, 2025.

**Fig. 1:**
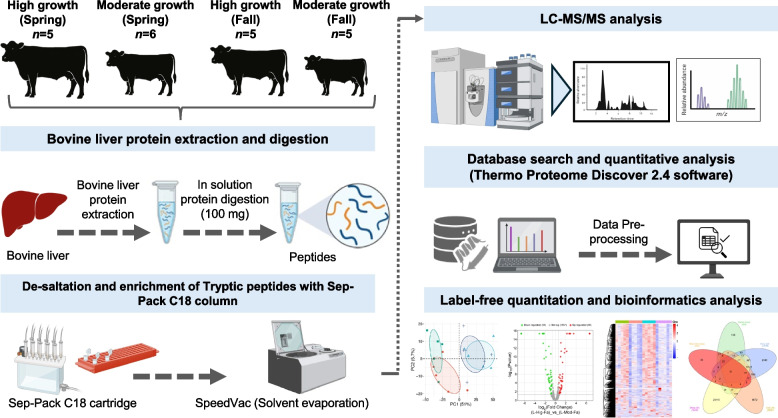
Workflow for bovine liver proteome analysis across growth and seasonal groups

This experimental design enabled the independent evaluation of sire growth potential and birth season effects on hepatic proteomic profiles under uniform post-weaning management. Groups for the liver proteomic analysis were denoted as 1) high growth-fall born (L-Hig-Fa), 2) high growth-spring born (L-Hig-Sp), 3) moderate growth-fall born (L-Mod-Fa), and 4) moderate growth-spring born (L-Mod-Sp; Fig. 1). Growth phenotype (high vs. moderate) and birth season (spring vs. fall) defined the experimental groups for proteomic analyses, whereas blocking and stratification procedures were applied only during feedlot management and performance evaluation and did not alter experimental group assignment. A subset of weaned (195 ± 13.4 days of age) steer calves (*n* = 5 or 6 steers/growth trait/calving season) raised at OSU RCRC South Range Unit (263 ± 12.2 kg) and North Range Unit (226 ± 13.4 kg) in 2023.

### Preconditioning and feedlot management

Newly weaned calves had access to prairie grass pastures and were provided with a concentrate supplement at 1.81 kg/steer/d. Decoquinate (Deccox, Zoetis Animal Health) was supplied at 0.65 mg/kg/steer/d as a top-dress for 23 consecutive days. *Amprolium* (Corid, Huvepharma, Peachtree City, GA, USA) was supplied via water according to label instructions using the 5-d protocol.

Following the preconditioning phase, steers were transported 22.5 km to the Willard Sparks Beef Research Center for the feedlot portion of the study. Calves were received and rested 24 h with access to *Cynodon dactylon* hay and ad libitum access to water before they were processed the next morning. At initial processing, calves received a growth-promoting implant (Component TE-S, Elanco Animal Health, Greenfield, IN, USA; Synovex Choice, Zoetis, Parsippany-Troy Hills, NJ, USA), clostridial (Vision 7 with SPUR, Merck Animal Health, Rahway, NJ, USA), an oral anthelmintic (Safeguard, Merck Animal Health), a *Fusobacterium necrophorum* bacterin (Fusoguard, Elanco Animal Health), and topical insecticide (Permectin II Animal and Premise Insecticide, Elanco Animal Health). Cattle were randomly assigned to 45 m^2^ soil-surfaced pens (5 steers/pen) and provided a standard starter diet at 0.45 kg/steer/d (Table 1).

**Table 1:** Ingredient composition and nutrient analysis of the diets fed to steers during the feedlot phase^a^

Item	Receiving	Transition^b^				Finishing^c^
		1	2	3	4	
Ingredient, % of DM						
Prairie hay	28.44	24.35	20.30	16.28	11.90	8.00
Sweet Bran	51.36	46.25	40.80	34.52	28.50	20.00
Rolled corn	15.00	24.40	33.90	44.20	54.60	62.00
Liquid supplement^d^	-	-	-	-	-	5.00
Mineral supplement^e^	5.20	5.00	5.00	5.00	5.00	5.00
Nutrient analysis (DM basis)						
Dry matter, %	72.40	73.18	74.40	75.89	77.35	78.45
Crude protein, %	18.08	15.45	14.84	14.12	13.44	13.32
TDN, %	70.14	71.79	74.03	76.36	78.79	82.59
NDF, %	38.30	34.73	31.07	27.24	23.26	18.44
ADF, %	21.31	18.03	15.79	13.50	11.07	8.76
NEm, Mcal/kg	1.45	1.47	1.54	1.62	1.31	1.81
NEg, Mcal/kg	0.90	0.95	1.01	1.07	1.13	1.21

^a^Analyzed and calculated by Servi-Tech Laboratories (Amarillo, TX, USA)

^b^Diet transitions from the receiving to finishing diet were executed over an average of 3 days per step

^c^Ractopamine hydrochloride (Optaflexx, Elanco, Greenfield, IN, USA) was included in the mineral supplement at 300 mg/animal/d for 28 consecutive days

^d^Liquid 10–10, Westway Feed Products, Tomball, TX, USA. Crude protein 10%; crude fat 10%; dry matter 63%

^e^The dry trace minearal premix supplement was formulated to contain (% DM basis): 40.0% ground corn, 29.6% limestone, 20.0% wheat middlings, 7.0% urea, 1.0% salt, 0.53% magnesium oxide, 0.51% zinc sulfate, 0.17% manganese oxide, 0.13% copper sulfate, 0.08% selenium premix (0.6%), 0.0037% cobalt carbonate, 0.32% vitamin A (30,000 IU/g), 0.10% vitamin E (500 IU/g), 0.009% vitamin D (30,000 IU/g), 0.20% tylosin (Tylan-40, Elanco Animal Health, Greenfield, IN) and 0.33% monensin (Rumensin- 90; Elanco Animal Health)

BWs used for performance analysis were collected at weaning, 60 d post-weaning (post-weaning), and harvest BWs were determined from the average of the day prior to and the day of harvest. Days on feed were also recorded for each growth × calving season group. After the 70-d individual feed intake data collection period, cattle were blocked by their sire growth EPD category and stratified by BW into small head pens (4 ± 1 steer/pen). Within stratification, heavy and light BW groups were derived to ensure steers were harvested at a similar target backfat thickness of 1.52 cm. Steers were administered a terminal growth-promoting implant (Component TE-S, Elanco; Animal Health Synovex Plus, Zoetis) 80 d prior to harvest. *Ractopamine hydrochloride* (Optaflexx, Elanco Animal Health) was included in the finishing diet for the last 28 DOF.

### Feed intake trial

During this portion of the study, a 70-d feed intake trial was conducted where individual animals' daily dry matter intake (DDMI) and BWs were routinely collected to determine feed conversion ratio (FCR; gain:feed) and average daily gain (ADG). Cattle were fed a roughage-based diet for 28 d, followed by a 4-step transition diet (Table 1) to acclimate steers to a high concentrate diet. Once cattle were in the step-up diet's second phase, they were weighed, randomly assigned to one of two partially covered (6.10 m) 362 m^2^ soil-surface pens, where Insentec Feeding Systems (Hokofarm Group, Marknesse, Netherlands) were housed. Body weights were collected on d 0, 14, 28, 42, 56, and 70. Feed intake data retrieved from the Insentec system was cleaned to ensure accurate and quality data was used for analysis. Daily feed intakes were averaged over the 70-d period. Predicted DMI and ADG were calculated using a multiple linear equation:

$$y= \mu + {\beta }_{w} {X}_{w}+{\beta }_{f} {X}_{f}$$

where $y$ is DMI or ADG, $\mu$ is the overall mean; ${X}_{w}$ is the mean metabolic weight (BW^0.75^) of the animal during the feed intake test period; and ${X}_{f}$ is the animal’s ADG or DDMI over the 70-d feed intake trial. The parameters ${\beta }_{w}$ and ${\beta }_{f}$ represent the regression coefficients for the effects of metabolic weight and ADG or feed intake, respectively. The residual portion would represent the deviation of the individual animal’s actual feed intake or ADG, subtracted from the predicted variables of the individual animal. Lower (negative) residual feed intake (RFI) values indicate steers that consume less feed than expected and are therefore more feed efficient, while higher (positive) RFI values indicate less efficient animals. Conversely, lower (negative) residual average daily gain (RADG) values indicate steers that gain less than expected and are less desirable, whereas positive RADG values reflect above expected growth.

### Liver biopsies

Steers were brought to the processing facilities the morning of liver biopsy collections on d 81 and following the 70-d feed intake trial. Liver biopsies were performed using the Wilson et al. protocol [24] by trained personnel, but in brief: The biopsy site was brushed clean, and an 11 cm × 11 cm area was clipped with a 0.1-cm surgical blade. The clipped area was cleaned in a circular motion, once each with a betadine solution and isopropyl alcohol-soaked gauze. Then 10 to 15 mL of Lidocaine (20 mg/mL) was administered between the 11^th^ and 12^th^ ribs, starting in the musculature, and ending in the subcutaneous tissue. The surgical area was cleaned again in circular motions, alternating between betadine solution and isopropyl-soaked gauze at least three times. A 1-cm incision was made between the 11^th^ and 12^th^ ribs with a #22 scalpel blade after ensuring the area was completely blocked. A 14-gauge, 15-cm Tru-Cut–style biopsy needle (Jorgensen Laboratories, Loveland, CO, USA) was introduced through the peritoneum and directed cranially and ventrally toward the animal’s contralateral (left) elbow. Liver tissue was collected by activating the biopsy mechanism upon hepatic entry, and the needle was withdrawn. Sampling was repeated until approximately 700 µg of liver tissue was obtained. Liver tissue samples were rinsed with phosphate-buffered saline, placed in sterile microcentrifuge tubes, and flash frozen immediately in a dry ice/alcohol mixture. Tissue samples were stored in a −80 °C freezer until further analysis. Incisions were closed with skin glue and sprayed with an adhesive bandage.

### Tissue preparation for proteomic analysis

The frozen bovine liver samples were homogenized using a Fisherbrand bead mill homogenizer (catalog No. 15-340-163). Each sample was transferred into Fisherbrand bulk tubes containing metal beads (catalog No. 15-340-151), followed by the addition of 500 µL of 8 mol/L urea prepared in 20 mmol/L HEPES buffer (pH 8.0) to facilitate protein lysis. Homogenization was performed for 1 min (speed: 6, cycles: 01) to ensure complete tissue disruption. Samples were immediately placed on ice to minimize potential degradation caused by heat generated during homogenization. The lysates were subsequently diluted with 20 mmol/L HEPES buffer to reduce the urea concentration below 2 mol/L, allowing accurate protein quantification using a NanoDrop™ One microvolume UV–Vis spectrophotometer (Thermo Fisher Scientific, IL, USA).

### Sample processing for bottom-up proteomics

Protein content was quantified by measuring absorbance at 280 nm using a NanoDrop™ One/OneC UV–Vis spectrophotometer (Thermo Fisher Scientific, IL, USA). For each sample, 100 µg of protein was subjected to enzymatic digestion with trypsin/Lys-C (V5071, Promega, USA). Digestion was performed in-solution, following the vendor’s recommendations to ensure efficient proteolysis. The resulting peptides were purified on C18 Sep-Pak Plus cartridges (Waters, Milford, MA, USA), dried under vacuum for approximately 8 h, and reconstituted in 200 µL of buffer A (0.1% formic acid). Peptide concentration was adjusted to 1.0 µg/µL, and 3 µg from each sample was used for downstream LC–MS/MS analysis.

### LC–MS/MS analysis

Chromatographic separation and peptide analyses were performed using a Dionex UltiMate™ 3000 Ultra-High-Performance Liquid Chromatography (UHPLC) system (Thermo Fisher Scientific, CA, USA) coupled to a Q Exactive™ HF-X Orbitrap mass spectrometer (Thermo Fisher Scientific, Waltham, MA, USA), as described previously [25, 26]. Peptides were separated on a Thermo EasySpray C18 analytical column (75 µm × 30 cm, 3 µm particle size, ES900, Germany) maintained at 55 °C. A linear gradient from 5% to 35% buffer B (0.1% formic acid in acetonitrile) was applied over 70 min at a flow rate of 350 nL/min, followed by column washing at 95% buffer B and re-equilibration at 5%. The total run time was 90 min.

All ionization and mass spectrometric acquisition parameters followed the same workflow described in these studies [25, 26]. Briefly, ionization was achieved via nano-electrospray using the integrated EasySpray emitter at 2.0 kV. The mass spectrometer was operated in positive ion mode with a capillary temperature of 320 °C and funnel radio frequency (RF) lens set to 40. Data-dependent acquisition (DDA) was carried out in Full MS-ddMS^2^ mode using a “top 15” method for precursor selection. Full MS scans were acquired at 60,000 resolution (*m*/*z* 200) with an automatic gain control (AGC) target of 3 × 10^6^ and a maximum injection time of 45 ms, over a scan range of *m*/*z* 350–2,000. MS/MS spectra were collected at 15,000 resolutions with an AGC target of 1 × 10^5^, maximum injection time of 30 ms, and an isolation window of 1.3 *m*/*z*. Higher-energy collisional dissociation (HCD) was performed with a normalized collision energy (NCE) of 30%. Dynamic exclusion was enabled for 30 s, with peptide match set to “preferred”, and isotope exclusion activated. Singly charged, unassigned, and ions with charge states ≥ 6 were excluded from fragmentation.

### Data analysis for bottom-up proteomics

Raw spectra were processed in Proteome Discoverer (PD) v2.4 (Thermo Fisher Scientific, San Jose, CA, USA) following the same data processing workflow described previously [25–27]. Peptide spectrum matches were searched against the UniProt Bos taurus reference proteome (TaxID: UP000009136) using the Sequest algorithm. Search parameters included trypsin cleavage specificity, allowance of up to two missed cleavages, precursor ion tolerance of 10 ppm, and fragment ion tolerance of 0.02 Da. Carbamidomethylation of cysteine residues (+57.0215 Da) was set as a fixed modification, while methionine oxidation (+15.9949 Da) was considered a variable modification. Results were filtered to a false discovery rate (FDR) of 1%. Label-free quantitation across samples was performed with the Minora algorithm integrated in PD 2.4.

### Performance and bioinformatics analysis

Metabolic BW and ADG for each individual steer were used for RFI and RADG determination by regressing BW on day of BW collections using the PROC REG procedure in SAS (SAS Inst. Inc., Version 9.4, Cary, NC, USA). The statistical analysis of BW, DOF, RFI, RADG, DMI, and FCR was performed using the MIXED procedure of SAS, with calving season, growth traits, and their interaction included as fixed effects, contemporary group served as the random statement, and animal was the experimental unit. Significance was declared at *P* ≤ 0.05 and tendencies at 0.05 < *P* ≤ 0.10.

Functional enrichment and pathway mapping of identified proteins were carried out using ShinyGO v0.85 [28, 29]. Data visualization was performed with a combination of open-access tools: SRplot for PCA, heat maps, and volcano plots [30]; InteractiVenn [31] and Venny 2.1.0 for Venn diagrams [32]; and Cytoscape for protein–protein interaction network construction [33]. Illustrative figures were designed using BioRender (https://app.biorender.com).

## Results

### Growth performance and efficiency data

Least-squares means for performance and efficiency data are presented in Table 2. The Hig steers tended to have heavier BWs (*P* = 0.07) at weaning than the Mod steers, and spring-born calves’ BWs (*P* = 0.01) were 37 kg heavier than the fall-born group. Post-weaning and harvest BWs (*P* = 0.01) for Hig steers were heavier than Mod steers, and the spring calving season group tended to have heavier BWs (*P* = 0.07) than the fall calving group. Fall-born groups required more DOF (*P* ≤ 0.01) to reach comparable harvest weights as spring-born calves. Dry matter intake (*P* = 0.09) tended to be greater in Hig steers than Mod steers, and spring-born calves’ DMI (*P* ≤ 0.01) was 4 kg greater than the fall-born calves. The fall calving season had an improved FCR (*P* < 0.01) of 0.05 kg compared to the spring calving season. These growth performance results provide physiological background information for the subsequent liver proteomic analysis.

**Table 2:** Effects of growth traits by calving season on body weight, efficiency metrics, and days to harvest

Item	Growth × Season group^1^				SEM^2^	*P-*value		
	L-Hig-Fa	L-Mod-Fa	L-Hig-Sp	L-Mod-Sp		Growth	Season	Growth × Season
BW, kg								
Wean	246	206	267	258	13.3	0.07	0.01	0.23
Post-weaning^3^	310	260	319	297	12.4	0.01	0.07	0.25
Harvest	705	662	698	672	13.5	0.01	0.93	0.52
Days on feed^4^	288	300	246	257	8.6	0.19	< 0.01	0.96
Feed intake trial^5^								
RFI, kg	0.25	0.15	0.24	−0.03	0.444	0.67	0.82	0.85
RADG, kg/d	−0.13	0.03	0.05	−0.15	0.116	0.87	0.97	0.11
DMI, kg	9.05	8.08	13.49	12.15	0.690	0.09	< 0.01	0.78
Feed conversion ratio	0.20	0.22	0.16	0.16	0.014	0.62	< 0.01	0.36

^a,b^Least-squares means followed by different superscripts within rows differ (*P* ≤ 0.05)

^1^Liver (L) was the tissue specimen collected for analysis. Hig-Fa: High growth trait-fall born calves; Mod-Fa: Moderate growth trait-fall born calves; Hig-Sp: High growth trait-spring born calves; Mod-Sp: Moderate growth trait-spring born calves

^2^Standard error of the mean; *n* = 5 or 6/growth × season group

^3^Post weaning = body weight collection 60 d post weaning

^4^Days on feed represents the time from weaning to harvest

^5^Efficiency and intake metrics were a result of a 70-d individual animal feed intake trial. *DMI* Dry matter intake, *ADG* Average daily gain, *RFI* Residual feed intake, *RADG* Residual average daily gain, *Feed conversion ratio* Gain:feed

### Quality assessment of bovine liver proteome profiling

**Fig. 2:**
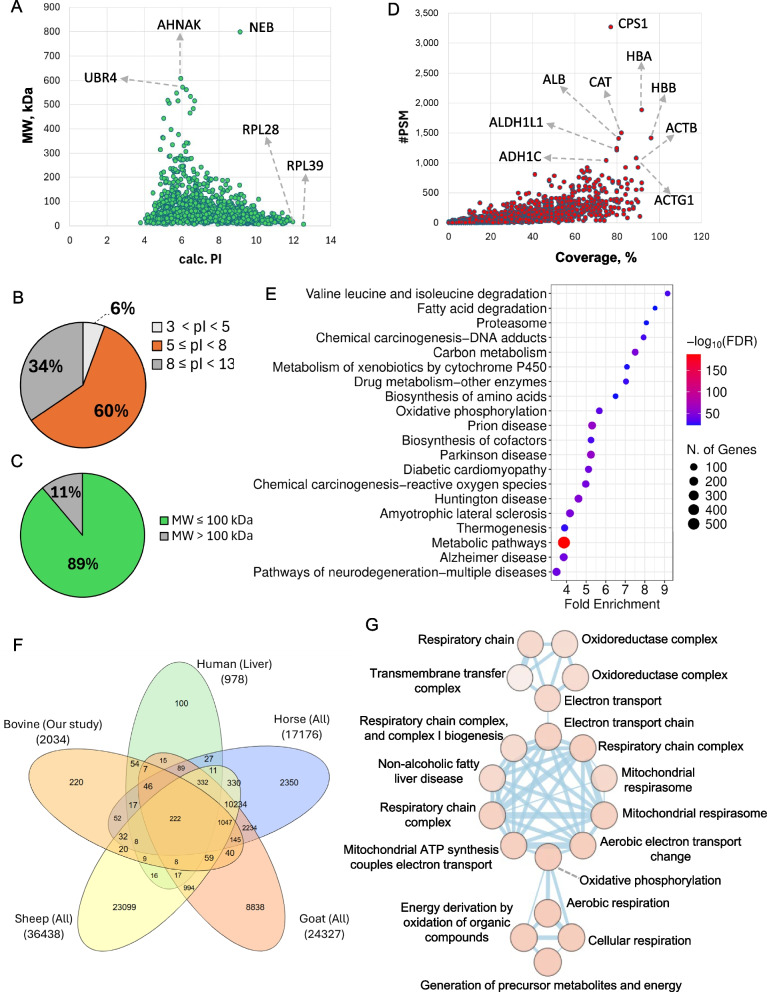
Global overview of the bovine liver proteome. **A** Scatter distribution of proteins based on theoretical molecular weight (MW) and isoelectric point (pI), showing clustering between 10–100 kDa and pI 5–8. **B** and **C** Pie chart distributions of MW and pI revealed that 89% of proteins were ≤ 100 kDa, with most proteins falling in the neutral-to-basic range. **D** Peptide spectral matches (PSMs) plotted against sequence coverage demonstrated broad dynamic range, with highly abundant proteins such as CPS1, ALB, ALDH1L1, ADH1C, CAT, HBA, HBB, and ACTB among the top hits. **E** KEGG pathway enrichment highlighted central hepatic functions, including amino acid and fatty acid metabolism, oxidative phosphorylation, and xenobiotic metabolism. **F** Venn diagram comparison of bovine liver proteins (*n* = 2,034) with human, horse, sheep, and goat proteomes identified 222 conserved proteins and 220 bovine-specific proteins. **G** Cytoscape network visualization of enriched functional modules showed three major clusters related to xenobiotic metabolism, mitochondrial respiration, and energy derivation pathways

Using a shotgun proteomics workflow, 2,133 proteins were identified from bovine liver samples across growth (high vs. moderate) and seasonal (spring vs. fall) groups (Table S1). The scatter distribution of identified proteins by theoretical molecular weight (MW) and isoelectric point (pI) revealed clustering between 10–100 kDa and pI 5–8, consistent with the expected physicochemical range of soluble hepatic proteins (Fig. 2A). Notably, several high-molecular-weight proteins such as nebulin (NEB), AHNAK, and UBR4 were confidently detected within the mid-pI range (6–8), demonstrating the instrument’s ability to capture large, complex structural proteins. In contrast, smaller ribosomal peptides including RPL28 and RPL39 were also identified, highlighting the wide dynamic range and sensitivity of the LC–MS/MS analysis used in this study.

Pie-chart distributions showed that 89% of proteins were ≤ 100 kDa (Fig. 2B) and ~ 60% had pI values between 5–8, with 34% in pI 8–13 and ~ 6% in pI 3–5 (Fig. 2C), indicating the predominance of neutral to mildly basic proteins.

Peptide spectral matches (PSMs) plotted against sequence coverage demonstrated a broad dynamic range of detection (Fig. 2D). Highly abundant hepatic proteins including carbamoyl-phosphate synthase 1 (CPS1), albumin (ALB), aldehyde dehydrogenase 1L1 (ALDH1L1), alcohol dehydrogenase 1 C (ADH1C), catalase (CAT), hemoglobin subunits (HBA, HBB), and actin isoforms (ACTB, ACTG1) served as internal benchmarks validating data quality. In contrast, low-abundance proteins such as glutathione peroxidase 3 (GPX3), cystathionine γ-lyase (CTH), and cytochrome P450 2B6 (CYP2B6) were detected with fewer spectral counts, reflecting minor yet functionally critical regulators of oxidative stress and detoxification [33]. Collectively, these metrics confirm that the dataset captures both highly abundant metabolic enzymes and lower-abundance regulatory proteins, providing a robust foundation for downstream enrichment analyses.

### Functional landscape of the bovine liver proteome

To evaluate the biological context of the identified proteins, we performed KEGG pathway enrichment on the complete dataset (Fig. 2E, Table S2). Strong associations were observed with core hepatic functions, including valine/leucine/isoleucine degradation, fatty acid degradation, carbon metabolism, oxidative phosphorylation, and xenobiotic metabolism by cytochrome P450. These results are consistent with the central metabolic role of the liver in nutrient catabolism, energy production, and detoxification.

Interestingly, enrichment also highlighted disease-relevant pathways such as chemical carcinogenesis (DNA adducts and ROS-related), proteasome, and neurodegenerative disorders (Alzheimer’s, Parkinson’s, Huntington’s, ALS, prion disease). These overlaps reflect the shared mitochondrial and redox machinery between hepatic metabolism and systemic pathophysiology, suggesting that the bovine liver proteome is not only central to growth and feed efficiency but also provides a valuable model for studying metabolic stress and disease susceptibility.

Cross-species comparison using all identified bovine liver proteins (*n* = 2,034) against human liver (*n* = 978), horse (*n* = 17,176), sheep (*n* = 36,438), and goat (*n* = 24,327) proteomes revealed a conserved mammalian liver core of 222 shared proteins, alongside 220 proteins uniquely detected in bovine liver (Fig. 2F, Table S3). These cattle-specific proteins were enriched in mitochondrial respiratory components, fatty acid β-oxidation enzymes, and xenobiotic-metabolizing enzymes, supporting ruminant-specific adaptations in energy and detoxification pathways. For this comparative analysis, reference proteomes were retrieved from UniProt for *Equus caballus* (horse; UniProt ID UP000002281), *Capra hircus* (goat; UniProt ID UP000291000), and *Ovis aries* (sheep; UniProt ID UP000664991). The human liver dataset (*Homo sapiens*; human) was obtained from the Human Protein Atlas (https://www.proteinatlas.org/) (liver-elevated protein subset) [34], while the *Bos taurus* (cow) proteome was generated in the current study (UniProt ID UP000009136). These datasets were used to construct the cross-species Venn diagram.

Functional analysis of the 220 bovine-specific proteins revealed distinct enrichment patterns (Fig. S1, Table S4). KEGG pathways (Fig. S1A) were enriched for cytochrome P450-mediated metabolism, chemical carcinogenesis (DNA adducts and ROS), porphyrin metabolism, fatty acid metabolism, and oxidative phosphorylation. GO Biological Processes (Fig. S1B) highlighted skeletal muscle contraction, ATP biosynthesis, respiratory chain complex assembly, aerobic respiration, and small-molecule catabolism. GO Molecular Functions (Fig. S1C) included oxidoreductase activity, glutathione transferase activity, NADH dehydrogenase activity, and peroxidase activity, underscoring redox balance. Finally, curated Reactome pathways (Fig. S1D) reinforced these findings, identifying modules in striated muscle contraction, TCA cycle regulation, TP53-controlled metabolic genes, fatty acid metabolism, and drug ADME.

Visualization in Cytoscape further revealed three interconnected enrichment modules (Fig. 2G, Table S5): (i) xenobiotic metabolism and drug detoxification, (ii) oxidative phosphorylation and mitochondrial electron transport, and (iii) respiratory/energy derivation pathways. Together, these results emphasize that the bovine liver proteome integrates both conserved mammalian functions and ruminant-specific adaptations, prioritizing detoxification, oxidative stress management, and mitochondrial energy production in support of systemic metabolic homeostasis.

### Growth- and season-dependent variation in liver proteome

**Fig. 3:**
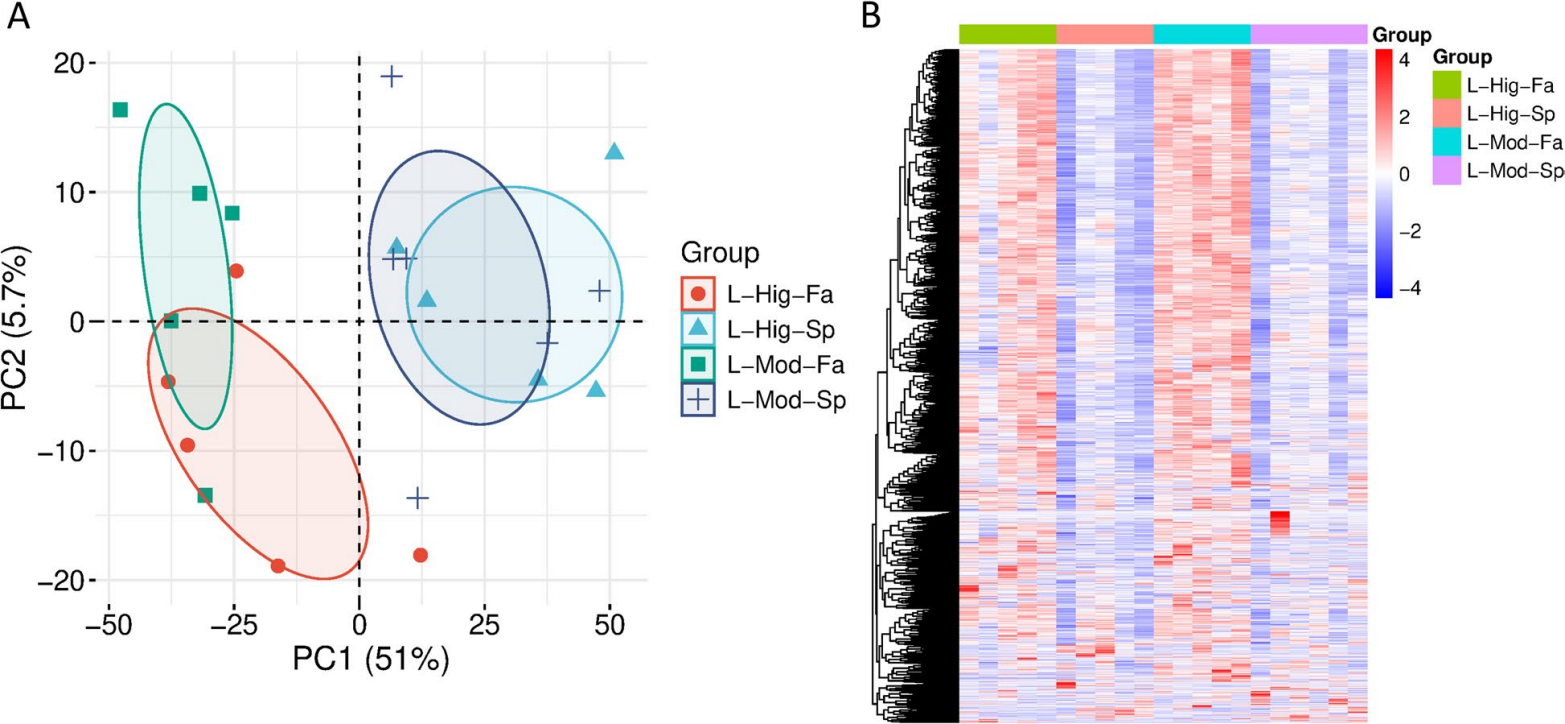
Multivariate analysis of bovine liver proteomes across growth and seasonal groups. **A** Principal component analysis (PCA) of quantified proteins revealed clear separation by birth season along PC1 (51% of variance), with spring-born calves (L-Hig-Sp, L-Mod-Sp) clustering distinctly from fall-born calves (L-Hig-Fa, L-Mod-Fa). PC2 (5.7% of variance) captured additional within-group variation, reflecting biological differences among individuals. **B** Hierarchical clustering analysis (HCA) further supported these patterns, showing distinct expression modules, with fall-born calves generally displaying higher protein abundance (red clusters) and spring-born calves showing lower abundance (blue clusters). Together, these results demonstrate that birth season/age exerts a stronger influence on the liver proteome than growth trait, though both factors contribute to distinct group-level signatures

To investigate the biological drivers of proteomic variation in bovine liver, we evaluated the combined influence of growth potential (high vs. moderate) and birth season (spring vs. fall). These contrasts primarily reflect environmental and physiological factors such as forage availability, temperature, and photoperiod. Principal component analysis (PCA) of quantified protein abundances revealed a clear separation of samples by birth season along PC1, which accounted for 51% of total variance (Fig. 3A). Spring-born calves (L-Hig-Sp, L-Mod-Sp) clustered distinctly from fall-born calves (L-Hig-Fa, L-Mod-Fa), indicating that the environmental conditions associated with each calving season alter the overall liver proteome. Principal Component 2 (5.7% variance) captured additional within-group variation, likely reflecting individual biological differences rather than systematic group effects.

Hierarchical clustering of all quantified proteins further supported these patterns (Fig. 3B). Distinct abundance modules were observed, with fall-born calves generally showing higher expression of proteins linked to stress and detoxification responses (red clusters), whereas spring-born calves displayed elevated abundance of proteins associated with efficient energy metabolism, mitochondrial function, and growth (blue clusters). These results align with the concept that spring-born calves experience more favorable nutritional and environmental conditions characterized by high-quality forage, moderate temperatures, and reduced oxidative or thermal stress in utero to weaning, leading to improved growth performance and metabolic efficiency. In contrast, fall-born calves encounter lower forage availability and greater environmental stress common to the region pre-weaning, prompting adaptive proteomic responses related to detoxification and oxidative balance.

Together, these multivariate analyses demonstrate that birth season, through environmental and nutritional differences, exerts a more substantial influence on the bovine liver proteome than inherent growth trait, although both factors contribute to distinct molecular signatures. These contrasts set the stage for subsequent differential-abundance testing, which identifies the specific proteins underlying the observed seasonal and growth-related differences.

### Differential protein abundance across growth and seasonal groups

**Fig. 4:**
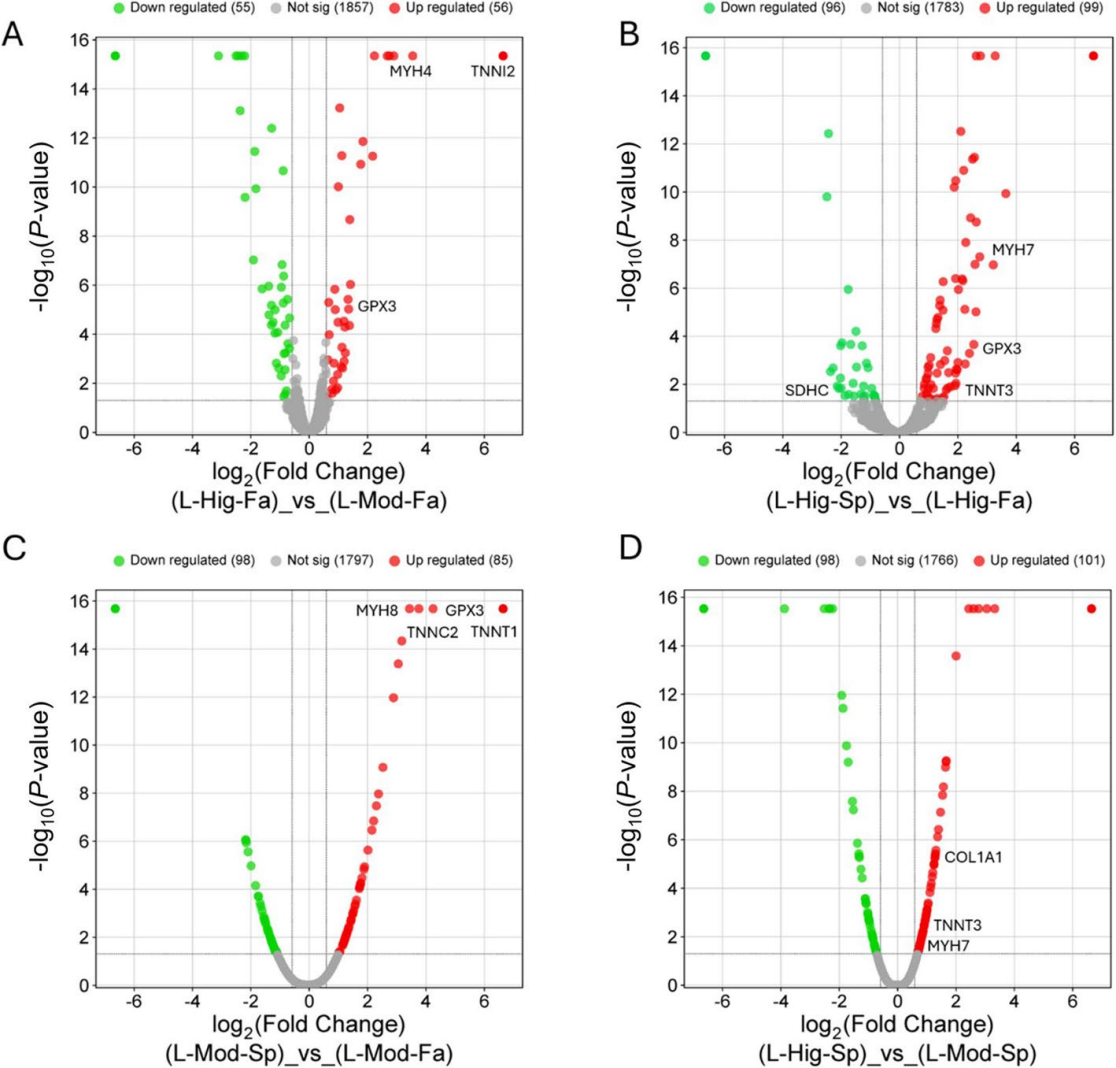
Differential protein abundance across growth and seasonal contrasts in bovine liver. Volcano plots display significantly upregulated (red) and downregulated (green) proteins between groups, with non-significant proteins in gray. **A** High-growth fall vs. moderate-growth fall (L-Hig-Fa vs. L-Mod-Fa) revealed 111 altered proteins, including upregulation of MYH4 and TNNI2 and downregulation of antioxidant GPX3. **B** High-growth spring vs. high-growth fall (L-Hig-Sp vs. L-Hig-Fa) showed 195 altered proteins, with enrichment of mitochondrial (SDHC) and structural proteins (MYH7, TNNT3), alongside modulation of GPX3. **C** Moderate-growth spring vs. moderate-growth fall (L-Mod-Sp vs. L-Mod-Fa) identified 183 altered proteins, including MYH8, TNNC2, and TNNT1 upregulated and GPX3 reduced. **D** High-growth spring vs. moderate-growth spring (L-Hig-Sp vs. L-Mod-Sp) showed the largest number of changes (199 proteins), with COL1A1, MYH7, and TNNT3 upregulated. Highlighted proteins represent top candidates associated with muscle structure, mitochondrial metabolism, and oxidative stress regulation

To identify proteins driving the separation patterns observed in PCA and clustering (Fig. 3), we performed pairwise differential abundance analyses across growth and seasonal contrasts (Fig. 4). Between high-growth and moderate-growth fall calves (L-Hig-Fa vs. L-Mod-Fa), a total of 111 proteins were differentially expressed, with 56 upregulated and 55 downregulated (Fig. 4A). Antioxidant enzyme GPX3 was reduced, suggesting an altered oxidative stress balance [35].

The comparison between high-growth spring and high-growth fall calves (L-Hig-Sp vs. L-Hig-Fa) identified the largest seasonal influence, with 195 altered proteins (99 upregulated, 96 downregulated; Fig. 4B). Spring-born calves exhibited enrichment of mitochondrial proteins, including SDHC, alongside structural proteins MYH7 and TNNT3 [36–38]. Interestingly, GPX3 was again modulated, pointing to consistent redox remodeling across seasonal contrasts [35].

In the moderate-growth groups, spring vs. fall comparison (L-Mod-Sp vs. L-Mod-Fa) revealed 183 differentially expressed proteins (85 upregulated, 98 downregulated) (Fig. 4C). Notably, contractile proteins MYH8, TNNC2, and TNNT1 were upregulated in spring-born calves, while GPX3 remained downregulated, underscoring the recurring involvement of antioxidant and structural pathways [35, 36].

Finally, the spring-born growth comparison (L-Hig-Sp vs. L-Mod-Sp) produced the largest contrast, with 199 proteins differentially expressed (101 upregulated, 98 downregulated) (Fig. 4D). Among them, extracellular matrix protein COL1A1, together with MYH7 and TNNT3, emerged as key drivers of growth-associated remodeling. Distinct clusters of up- and downregulated proteins were observed in each contrast, reinforcing that both growth potential and birth season (reflecting environmental and nutritional conditions) remodel the hepatic proteome. Proteins highlighted across multiple contrasts (e.g., MYH4, MYH7, MYH8, TNNI2, TNNT1, TNNT3, TNNC2, GPX3, COL1A1, SDHC) represent consistent markers of growth, structural development, mitochondrial metabolism, and oxidative balance. Together, these results indicate that while growth trajectory modifies the liver proteome, birth season exerts the stronger systemic influence, with both factors converging on shared molecular themes of muscle contractility, mitochondrial metabolism, extracellular matrix organization, and redox regulation.

### Condition-specific differential expression and functional insights

**Fig. 5:**
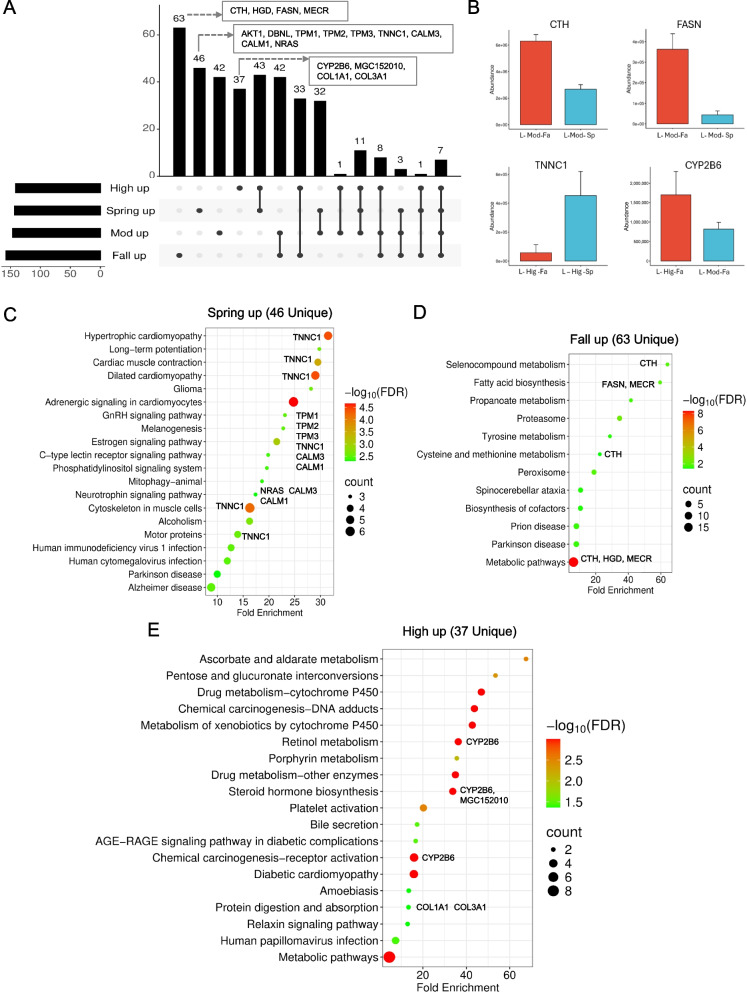
Core growth- and season-associated proteins in bovine liver. **A** UpSet plot showing overlap of up-regulated proteins across growth (High vs. Moderate) and seasonal (Spring vs. Fall) contrasts. Proteins are categorized by condition-specific regulations (High up, Moderate up, Spring up, Fall up), with intersection bars indicating shared proteins across multiple contrasts. **B** Representative abundance profiles of some selected proteins (CTH, FASN, TNNC1, CYP2B6) illustrating condition-specific regulation across growth and seasonal contrasts and their functional relevance to muscle contractility, lipid metabolism, and oxidative balance associated with beef quality traits. **C** KEGG pathway enrichment analysis of 46 proteins uniquely up-regulated in spring, highlighting pathways related to muscle contraction, adrenergic signaling in cardiomyocytes, cytoskeletal organization, and motor proteins. **D** KEGG pathway enrichment analysis of 63 proteins uniquely up-regulated in fall, revealing enrichment for metabolic pathways including fatty acid biosynthesis, amino acid metabolism, proteasome function, and redox-associated processes. **E** KEGG pathway enrichment analysis of 37 proteins uniquely up-regulated in High-growth animals and down-regulated in Moderate-growth animals, emphasizing xenobiotic metabolism, cytochrome P450–mediated detoxification, steroid hormone biosynthesis, and carbohydrate metabolism

To further disentangle the proteomic effects of growth and season, we constructed UpSet plots to track the overlap and uniqueness of differentially expressed proteins (DEPs) across groups (Fig. 5A). Distinct clusters of up- and downregulated proteins revealed that fall-associated downregulation corresponded strongly with spring-associated upregulation, and vice versa, highlighting opposite proteomic remodeling between calves born in contrasting environmental conditions.

KEGG pathway enrichment of these condition-specific DEPs revealed biologically coherent modules (Fig. 5C–E, Table S6). Proteins downregulated in fall but upregulated in spring were enriched in calcium-binding and contractile proteins (TNNC1, CALM1, CALM3, NRAS), implicating cytoskeletal remodeling and muscle tenderness pathways [39–41]. Conversely, spring-downregulated but fall-upregulated proteins mapped to fatty-acid biosynthesis and amino-acid metabolism (FASN, MECR, CTH, DCC, HGD), which are central to lipid turnover, antioxidant balance, and overall metabolic adaptation [42].

Among these, FASN (fatty-acid synthase) catalyzes de novo fatty acid synthesis, and in the liver it contributes to triglyceride production and lipid export via very low-density lipoproteins, thereby influencing systemic fatty acid availability that can be used for intramuscular fat storage when nutrients are present in excess [43]; MECR participates in mitochondrial fatty-acid elongation, linking energy metabolism with redox regulation; CTH (cystathionine-γ-lyase) modulates hydrogen-sulfide-dependent antioxidant and anti-inflammatory responses in bovine tissues [42]. Decellularized cancellous bone-related structural component (DCC) represents ECM remodeling processes associated with improved biocompatibility and osteogenic potential [44], and HGD (homogentisate 1,2-dioxygenase) catalyzes tyrosine catabolism, with bovine *HGD* polymorphisms linked to meat tenderness and shear-force traits [45].

Growth-specific contrasts (high vs. moderate) further highlighted enrichment in detoxification and steroid/retinol metabolism pathways, driven by proteins such as CYP2B6 and MGC152010, consistent with hepatic xenobiotic metabolism and previous bovine hepatocyte studies [46]. These metabolic and redox-associated proteins likely operate under the control of seasonally and growth-modulated regulatory networks. Recent multi-tissue epigenomic mapping of the bovine genome revealed extensive tissue-specific regulatory elements shaping transcriptional and post-transcriptional responses in the liver, including pathways involved in lipid turnover, oxidative stress, and xenobiotic metabolism [47].

To illustrate these findings, abundance profiles of representative proteins were examined (Fig. 5B). Cystathionine-γ-lyase and FASN were elevated in fall-born cattle, suggesting enhanced antioxidant and lipogenic capacity under cooler, forage-limited conditions, while TNNC1 expression was markedly higher in spring-born cattle, aligning with improved muscle contractility and growth efficiency during favorable nutritional periods. The expression of CYP2B6 was strongly growth-dependent, reflecting a role in hepatic detoxification. Together, these analyses demonstrate that bovine liver proteomic remodeling integrates growth rate and seasonal factors through condition-specific regulation of lipid metabolism, redox balance, and contractile machinery molecular adaptations that likely contribute to differences in lipid biosynthesis for energy storage intramuscularly and oxidative stability.

## Discussion

This study provides a comprehensive proteomic characterization of bovine liver with explicit consideration of two major biological factors: birth season as an environmental and developmental factor (reflecting variation in forage quality, temperature, and photoperiod across seasons) and growth trait as an intrinsic trait (high vs. moderate growth). Clarifying these distinctions is essential: “birth season” captures early-life environmental influences on hepatic physiology, whereas “growth” represents genetically and metabolically driven differences in feed efficiency and energy utilization. Using a shotgun, label-free proteomics approach in Angus and Angus-cross steer calves from a single ranch origin, we systematically mapped how these factors shape the hepatic proteome post-feedlot entry under a uniform diet.

### Performance outcomes

Calving season differences for weaning BWs suggest external environmental, forage quality, and availability directly influence fetal and pre-wean growth. Spring calving coincides with warm season grass emergence [48], aligning peak forage growth with maximal dam nutrient demand for milk, body condition, and rebreeding [49]. Fall calving cows graze cool season grasses during February to March growth, before their decline in nutritional value in September to November [49, 50]. In this study, spring calves were born between February and March, allowing dams to have access to higher quality forages during peak lactation when nutrient requirements are their highest. Fall calving dams, by contrast, were in late gestation during the drought season in Western Payne County, OK (August to September), facing declining forage quality when calves began consuming more dormant winter grasses and a declining milk supply. Similarities between harvest BWs are because of the additional DOF required to achieve a common backfat thickness. During the preconditioning period, steers were provided a restricted energy high protein-based supplement and free access to moderate quality *Cynodon dactylon* forage. The supplement allowed principal tissues such as muscle, adipose, and bone to be provided for energy allocation towards maintenance first and remaining energy towards gain (growth). Adequate dietary supply of energy and protein is critical for growing animals, even in nutritionally restricted animals that will benefit from compensatory gain when excess net energy is provided. After energy is consumed for maintenance by both product and support tissues, energy is moved into metabolic pathways that result in product formation and accretion, explaining the growth trait differences [51]. Dry matter intake (DMI) is driven by the demand to meet requirements for maintenance and growth of an animal and is determined by many factors, such as physiological limits determined by rumen fill, ruminal volatile fatty acid (VFA) concentrations, ruminal pH, and body fat [52]. Adequate nutrient availability early in life likely enabled spring-born calves to develop visceral tissues at a greater rate, supporting higher intake capacity, whereas fall-born calves may not have achieved the same level of digestive organ development [11, 53, 54]. Increased DMI is commonly associated with enlarged visceral organs that exhibit high metabolic activity [53], thereby elevating digestive energy expenditure and maintenance requirements. Thus, differences in DMI between the calving seasons may reflect underlying energetic demands associated with visceral tissue development.

### Birth season effects

Only a modest subset of proteins was differentially expressed between spring- and fall-born calves, suggesting that the hepatic proteome is largely conserved. However, proteins that did differ were enriched in immune, and stress-response pathways, indicating that early environmental exposures leave detectable proteomic signatures. Immune-modulating proteins such as proteasome subunit beta type-2 (PSMB2) and SERPINA3-4 were downregulated in fall-born calves, while complement component C4 showed the opposite regulation, suggesting adaptive immune tuning across environmental gradients. KEGG analysis further supported seasonal influences, highlighting cysteine and methionine metabolism, fatty acid biosynthesis, and glutathione metabolism, key pathways involved in antioxidant defense [42].

### Growth trait effects

Growth trait exerted a more substantial influence on metabolic remodeling, particularly in amino acid, fatty acid, and glucose pathways. Enzymes such as PM20D1 and AKT1 were upregulated in high-growth calves, consistent with increased energy turnover and enhanced metabolic signaling [55, 56]. Immune-linked proteins DBNL, CTSS, and NLRX1 were also elevated, suggesting compensatory immunometabolic responses to rapid growth demands [57–59].

### Joint regulation by growth and season

A subset of 14 proteins, including BAG6, ADA, and COX7A2L, was co-regulated by both growth and season. These proteins bridge the connection between immune modulation and mitochondrial assembly, underscoring the liver’s role in integrating energy metabolism and immune readiness [60–62]. Additional detections of MYL1, and TNNC2, typically muscle-associated proteins, suggest systemic liver–muscle crosstalk during growth adaptation [46, 63, 64].

### Pathway-level integration and beef quality traits

By integrating differential expression with UpSet plots and KEGG analyses (Fig. 5), we observed reciprocal regulation between spring and fall groups: proteins downregulated in fall were frequently upregulated in spring, and vice versa, reflecting environmental plasticity in hepatic metabolism. Importantly, many seasonal proteins were mapped to pathways that influence beef quality traits. Fatty acid biosynthesis (FASN) and propanoate metabolism drive intramuscular fat, while cysteine and methionine metabolism (CTH) modulate sulfur-containing volatiles and antioxidant defense. Glutathione metabolism supports oxidative stability and shelf life, whereas collagen signaling (COL1A1, COL3A1) influences connective tissue remodeling and tenderness. Selective expression of FASN, CTH, and CYP2B6, each linked to distinct KEGG pathways and detoxification processes [46], underscores condition-specific proteomic remodeling. These metabolic patterns align with emerging epigenomic evidence showing that liver transcriptional networks are shaped by regulatory elements responsive to environmental and developmental cues [47]. Together, these data suggest that birth season and growth trait jointly tune hepatic metabolism to balance energy efficiency, redox homeostasis, and intramuscular energy storage.

Taken together, the findings of this study demonstrate that birth season and intrinsic growth potential converge on shared hepatic pathways governing energy metabolism, redox balance, immune regulation, and structural remodeling. Birth season exerted the dominant influence on the liver proteome, reflecting early-life environmental exposure associated with forage availability, temperature, and photoperiod, whereas growth trait more selectively modulated immune, detoxification, and metabolic efficiency pathways. Importantly, integration of growth performance metrics with proteomic remodeling indicates that observed differences in body weight, feed intake, and days on feed are underpinned by coordinated regulation of mitochondrial metabolism, lipid biosynthesis, antioxidant defense, and extracellular matrix organization. The reciprocal regulation observed between spring- and fall-born calves highlights the metabolic plasticity of the bovine liver and underscores its role as a central environmental and metabolic sensor that integrates genetic growth potential with early-life conditions to shape downstream growth performance and beef quality traits.

## Conclusion

This study presents the first comparative proteomic map of bovine liver explicitly partitioning the effects of birth season (environment) and growth trait. From over 2,100 proteins, we defined both a conserved mammalian liver core and cattle-specific signatures enriched in mitochondrial respiration, β-oxidation, and detoxification pathways. Birth season exerted the stronger influence overall, shaping oxidative stress, circadian, and metabolic pathways, while growth trait more selectively engaged immune and redox-related modules. Importantly, the reciprocal regulation of key pathways between spring and fall-born calves, combined with selective expression of FASN, CTH, and CYP2B6, provides a molecular explanation for seasonal differences in fat storage intramuscular and oxidative stability.

Collectively, these findings establish the bovine liver proteome as a metabolic hub and environmental sensor that integrates intrinsic growth potential with extrinsic seasonal factors. Beyond advancing ruminant biology, this work identifies candidate biomarkers and pathways that can begin to inform genetic selection, nutritional management, and precision strategies.

## Supplementary Information

Additional file 1: Fig. S1. Functional enrichment of bovine liver–specific proteins unique to our dataset.

Additional file 2: Table S1. List of proteins identified by LC–MS/MS analysis.

Additional file 3: Table S2. Gene ontologydata supporting Fig. 2E.

Additional file 4: Table S3. Genes used for constructing the Venn diagram.

Additional file 5: Table S4. Gene ontologydata supporting Fig. S1.

Additional file 6: Table S5. Functional enrichment map data.

Additional file 7: Table S6. Gene ontologydata supporting Fig. 5.

## Data Availability

All LC–MS/MS RAW files and results files can be found at the MassIVE database (https://massive.ucsd.edu) under the following accession MSV000099692.

## Abbreviations

ADF: Acid detergent fiber
ADG: Average daily gain
AI: Artificial insemination
ATP: Adenosine triphosphate
BW: Body weight
CIDR: Controlled internal drug release
DDA: Data-dependent acquisition
DDMI: Daily dry matter intake
DEP: Differentially expressed protein
DM: Dry matter
DMI: Dry matter intake
DNA: Deoxyribonucleic acid
DOF: Days on feed
EPD: Expected progeny difference
FCR: Feed conversion ratio
FDR: False discovery rate
HCD: High-energy collisional dissociation
HEPES: 4-(2-Hydroxyethyl)-1-piperazineethanesulfonic acid
Hig: High growth
LC–MS/MS: Liquid chromatography–mass spectrometry/mass spectrometry
Mod: Moderate growth
MW: Molecular weight
NADH: Nicotinamide adenine dinucleotide hydrogen
NCE: Normalized collision energy
NDF: Neutral detergent fiber
NEg: Net energy for growth
NEm: Net energy for maintenance
OSU RCRC: Oklahoma State University Range Cow Research Center
PCA: Principle component analysis
PSM: Peptide spectral matches
pI: Isoelectric point
RADG: Residual average daily gain
RFI: Residual feed intake
TCA: Tricarboxylic acid
TDN: Total digestible nutrients
VFA: Volatile fatty acids
YW: Yearling weight
