# Supplementary material for: Comparative liver proteome analysis of feedlot steer calves reveals growth trait-specific pathways influenced by calving season
Source: J Anim Sci Biotechnol. 2026 Apr 3;17:56. doi: 10.1186/s40104-026-01370-6 (PMC13047832; doi:10.1186/s40104-026-01370-6)
Supplement: Supplementary file 1 — Additional file 1: Fig. S1. Functional enrichment of bovine liver–specific proteins unique to our dataset. [file 40104_2026_1370_MOESM1_ESM.pdf]

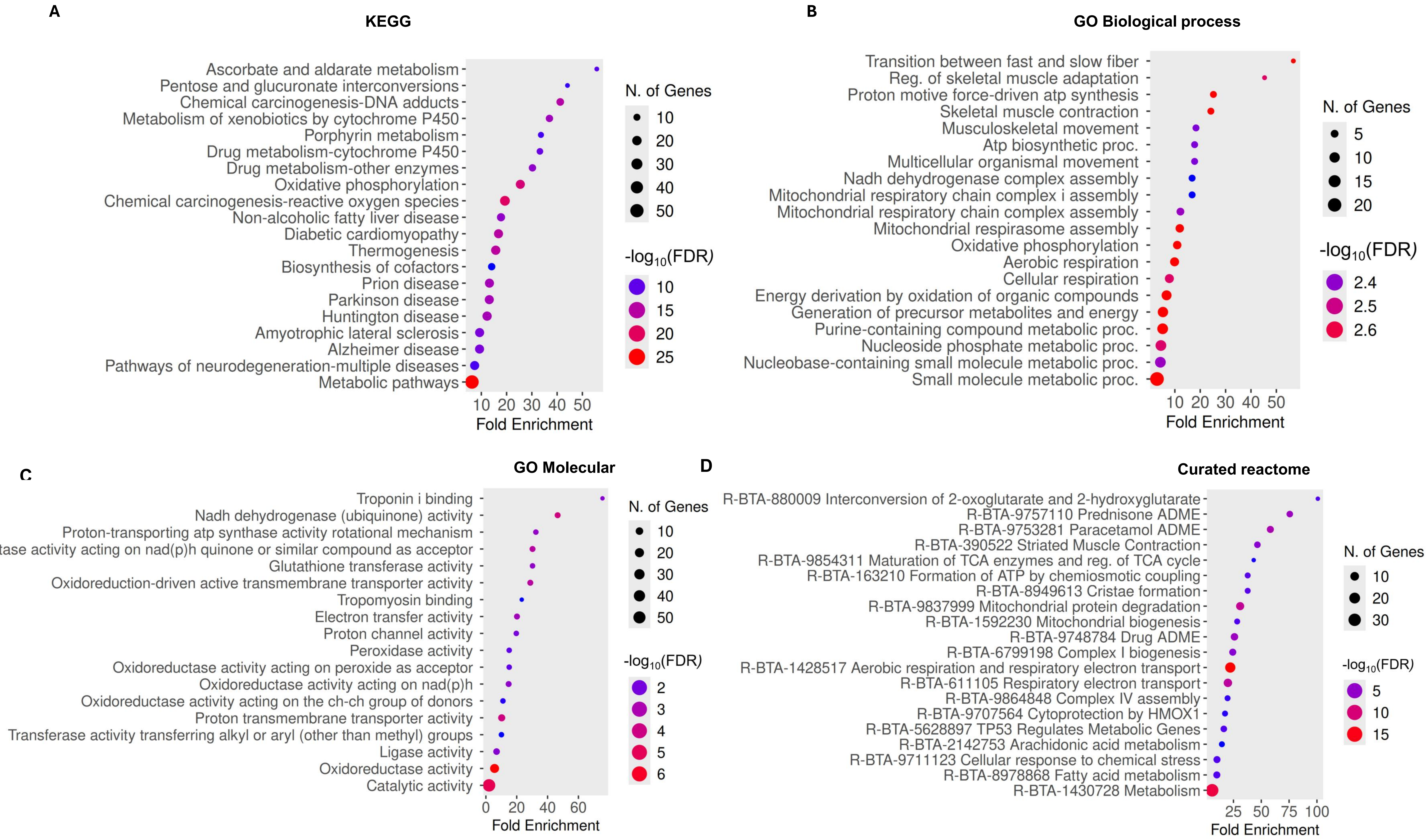

Supplementary Figure 1. Functional enrichment of bovine liver–specific proteins unique to our dataset (n = 220).
(A) KEGG pathway enrichment analysis revealed significant associations with metabolic and detoxification functions, including cytochrome P450–mediated metabolism, oxidative phosphorylation, glutathione metabolism, and multiple disease pathways. (B) Gene Ontology (GO) Biological Process terms showed enrichment for skeletal muscle contraction, mitochondrial respiratory chain complex assembly, oxidative phosphorylation, ATP biosynthesis, and multicellular movement, highlighting links between energy metabolism and structural remodeling. (C) GO Molecular Function analysis identified enrichment for oxidoreductase activity, NADH dehydrogenase activity, glutathione transferase activity, and proton transmembrane transporter activity, indicating strong roles in redox balance and electron transport. (D) Curated Reactome pathway analysis emphasized mitochondrial protein degradation, ATP generation, drug metabolism (ADME modules), mitochondrial biogenesis, and striated muscle contraction, underscoring the integration of detoxification, mitochondrial energetics, and structural functions.
